# Assessment of 1p/19q deletion by flourescence insitu hybridization (FISH) in glioma patients from Andhrapradesh

**DOI:** 10.1186/1755-8166-7-S1-P56

**Published:** 2014-01-21

**Authors:** Eppa Kavitha, Iravathy Goud, Swarna Latha, Meenakshi Swain, Michelle De Paude, Tejal Modi, Anuradha, Ravi V, Sakina Aneeb, Adi Maha Lakshmi M, Vijayanand Reddy P

**Affiliations:** 1 Molecular Biology and Cytogenetics Department, Apollo Health City Building, Apollo Hospitals, Jubilee Hills, Hyderabad, India; 2 Department of Histopathology, Apollo Hospitals, Jubilee Hills, Hyderabad, India; 3 Department of Oncology, Apollo Hospitals, Jubilee Hills, Hyderabad, India

## Background

Oligodendroglial tumors represent approximately 4-7% of all gliomas, however, in some series the incidence has been reported to be as high as 10-20% due to improved histological appreciation and recently recognized molecular signatures. The discovery of 1p and 19q chromosomal arms deletion in glial tumors influences both more objective diagnosis and more accurate prediction of chemotherapy response. As a result an attempt has been made to detect deletion using fluorescence in-situ hybridization (FISH) and to determine its prognostic value in a cohort of glial tumor patients from Andhra pradesh.

## Materials and methods

FISH was performed on 66 FFPE tissue sections by using Vyis LSI 1p36/LSI 1q25 and LSI 19p13/LSI 19q13 dual coloured FISH probe sets. Signals were scored from at least 150-250 non-overlapping, intact nuclei.

## Results

Simultaneous occurrence of both 1p and 19q deletions was observed in (21/35) 60% of oligodendroglyomas which included (8/21) 38% of grade II and (13/21) 61.9% of grade III. Isolated 19q deletion was seen in (1/21) 4.76% & lone 1p loss was not observed in oligodendroglyomas. In Mixed Oligoastrocytomas combined 1p/19q loss was observed in (7/16)43.75% cases, including one grade II and 6 grade III tumors and 1/16 (6.25%) showed isolated 1p loss & 19q deletion. This disorder was not observed in astrocytomas. The oligodendroglial phenotype was found to be significantly associated with a loss of 1p (P<0.05), a loss of 19q (P<0.05) and a combined loss of 1p and 19q (P< 0.05). Frontal location of a tumor occurred to be a statistically significant factor unfavourable for prognosis, p<0.05.

## Conclusion

In the work presented the FISH was successfully applied to identify deletion 1p/19q. Its incidence depends on the type of diagnosed glioma. Deletions also have prognostic significance in the test group what constitutes the basis for inclusion of determining deletion 1p/19q into diagnostic and treatment algorithm in gliomas.

